# A rare case of chondroblastoma involving the distal phalanx of the ring finger

**DOI:** 10.1016/j.radcr.2023.04.024

**Published:** 2023-05-11

**Authors:** Ethan Radzinsky, Cyrus Bateni, Raminta Theriault, Steven W Thorpe, Jasjeet Bindra

**Affiliations:** a Department of Radiology, University of California Davis School of Medicine, 4860 Y St. Suite 3100 Sacramento, CA 95817, USA; b Department of Orthopedic Surgery, University of California Davis School of Medicine, CA, USA

**Keywords:** Chondroblastoma, Hand, Phalanx, Magnetic resonance imaging

## Abstract

Chondroblastoma, a rare benign bone tumor, is typically found in the epiphysis of long bones, with hand involvement being particularly uncommon. We present a case of an 11-year-old female with chondroblastoma involving the fourth distal phalanx of the hand. Imaging revealed a lytic, expansile lesion with sclerotic margins and no soft tissue component. A preoperative differential diagnosis included intraosseous glomus tumor, epidermal inclusion cyst, enchondroma, and chronic infection. The patient underwent open surgical biopsy and curettage for both diagnostic and treatment purpose. The final histopathologic diagnosis was chondroblastoma.

## Introduction

Chondroblastoma is a rare, benign neoplasm accounting for approximately 1% of all primary bone tumors and 9% of all benign bone tumors [1,2]. It was initially described in 1928 as a “calcifying giant cell tumor” by Ewing and later termed “Benign Chondroblastoma” by Jaffe and Lichtenstein in 1942 due to its cartilaginous origin [3]. This tumor is frequently encountered in adolescents of mean age 15 years, and affects males twice as much as females [2].

Although chondroblastoma can arise in various bones, it is most commonly found in the epiphysis of long bones, with the proximal humerus, proximal tibia, and proximal and distal femur being the most frequent sites [4]. Involvement of the hands is rare [5], [6], [7], [8], [9]. One series identified only 3 cases of chondroblastoma in the hands out of 4680 bone tumors, affecting the metacarpal, triquetrum, and the phalanx [9]. To our knowledge, there have been seven reports of chondroblastoma involving a phalanx in the hand [7,[9], [10], [11], [12], [13], [14]]. Clinical manifestations include pain, local swelling, effusion, and limited range of motion at the adjacent joint [15].

Radiological imaging plays a crucial role in the diagnosis and management of chondroblastoma. Pathognomonic imaging findings of this entity include classic location, geographic and sclerotic margins, and cartilaginous matrix [16]. Advanced imaging modalities, such as computed tomography and magnetic resonance imaging (MRI), can further delineate tumor characteristics and assist in surgical planning. The rarity of chondroblastoma of the hand, along with the similar appearance to other bone tumors on imaging, poses challenges in accurate diagnosis [17].

The standard treatment typically consists of surgical intervention with curettage and bone grafting or cementation [6]. In this case report, we present a rare instance of chondroblastoma involving the fourth distal phalanx of the hand. By sharing this case, we aim to improve diagnostic accuracy and increase awareness of the key radiological findings that can help differentiate chondroblastoma from other bone tumors in atypical locations.

## Case report

**Fig. 1:**
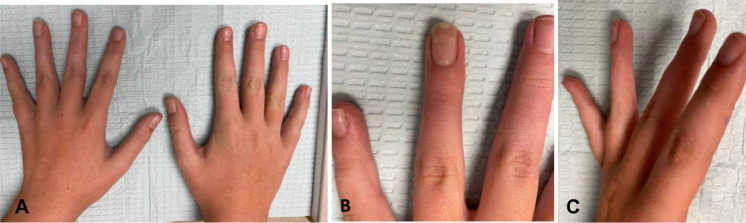
Clinical photographs of the patient at initial presentation showing (A) frontal view of the hands, (B) frontal and (C) lateral views centered on the left fourth digit.

An 11-year-old female with otherwise no past medical history presented to our institution with pain of the left ring finger distal phalanx 4 months after a minor injury. Prior to this incident the patient did not have any finger pain nor any mass or swelling. Given persistent symptoms the patient and parents decided to seek medical attention. History was negative for any fevers, chills, or weight loss. Physical exam did not reveal any motor deficit, swelling, or erythema, but there was tenderness to palpation over the nail bed (Fig. 1).

## Imaging

**Fig. 2:**
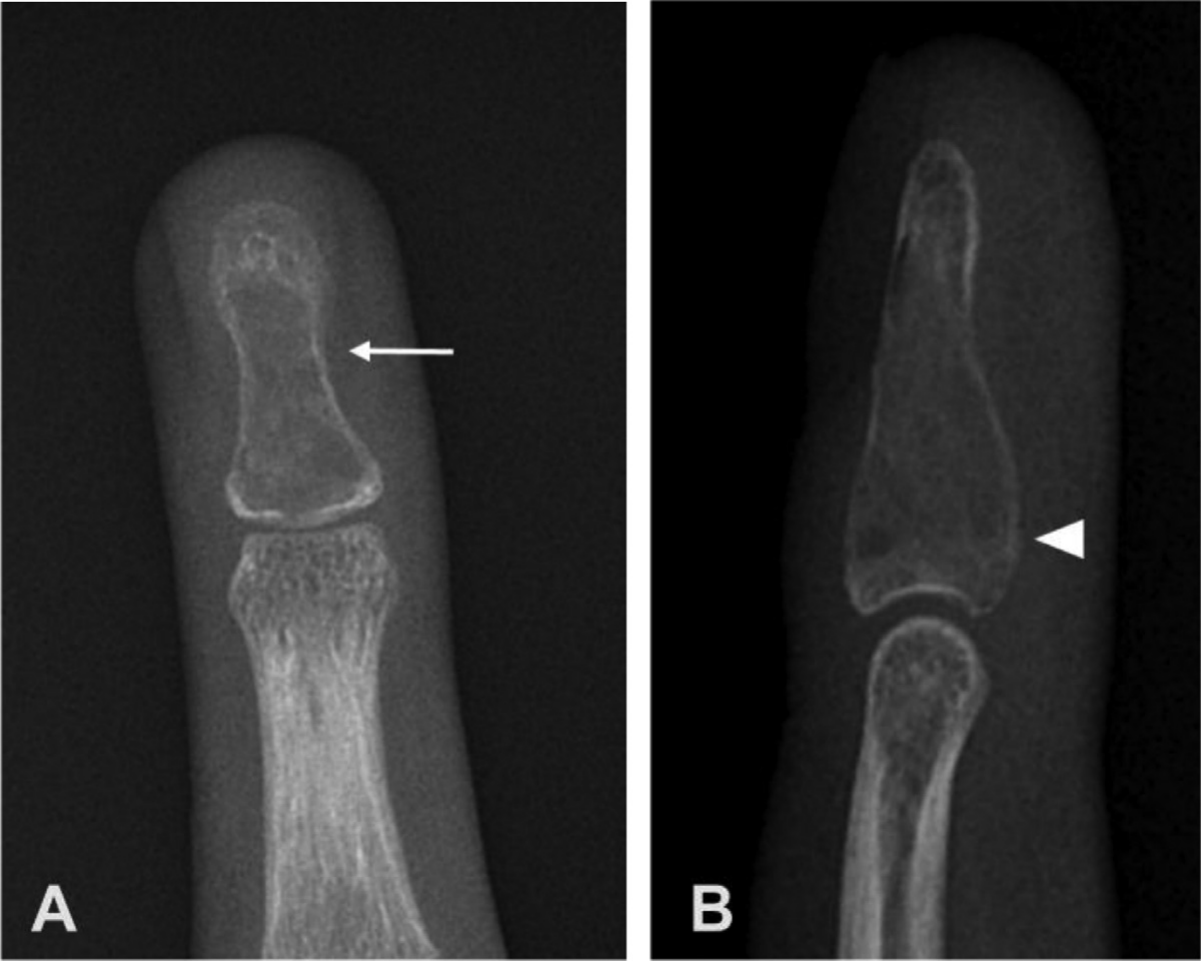
(A) PA and (B) lateral radiograph of the left hand showing a focal lytic, expansile lesion (white arrow) in fourth distal phalanx with minimal adjacent periostitis (arrowhead) and minimal fourth digit soft tissue swelling.

**Fig. 3:**
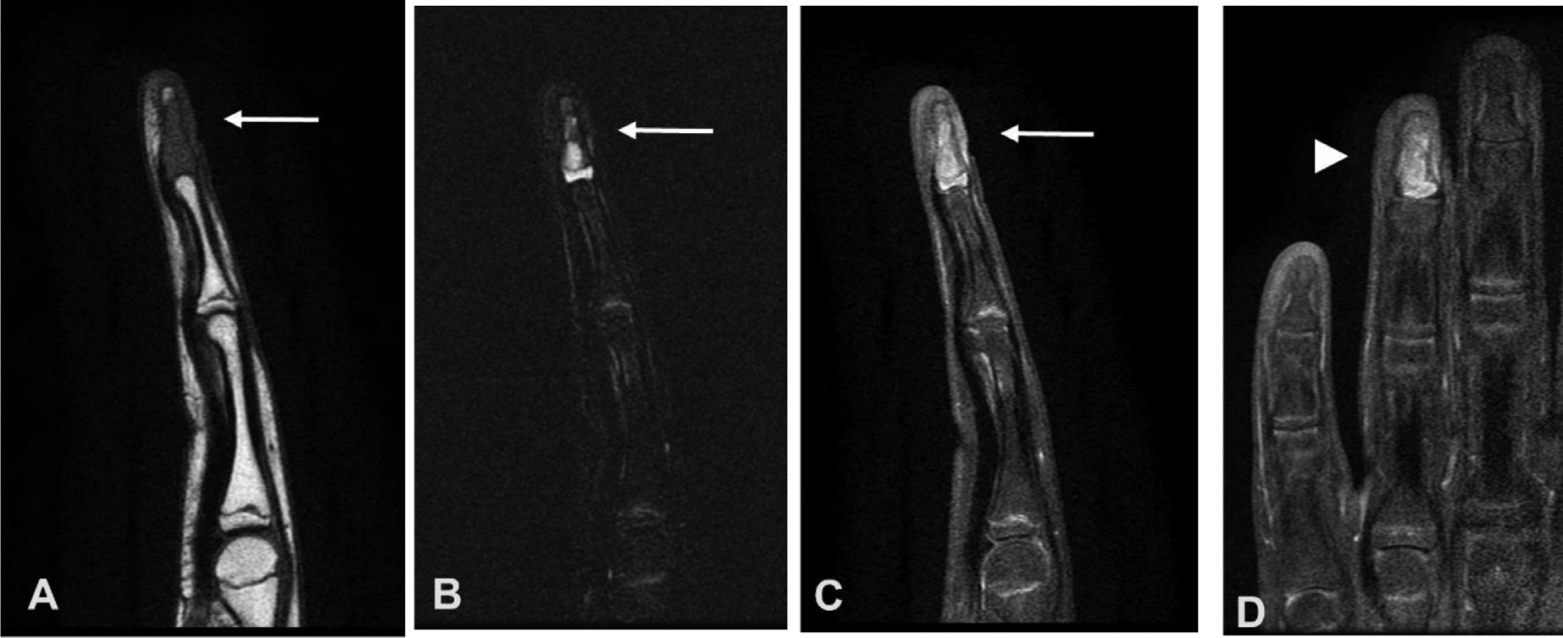
(A) Sagittal T1-weighted nonfat-suppressed MRI of the left hand demonstrates a hypointense lesion (arrow) involving the fourth digit distal phalanx, with slight sparing of the tuft and no cortical disruption. (B) Sagittal T2-weighted fat saturated and (C) T1-weighted fat saturated post contrast MRI of the left hand demonstrates heterogeneous increased signal intensity involving the bone (arrow) without abnormal soft tissue signal. (D) Coronal T1-weighted fat-saturated postcontrast images of the third through fifth digits demonstrates mild enhancement of the lesion (arrow head).

Due to the patient's symptoms, a radiograph of the hand was obtained (Fig. 2) and showed a lytic, expansile lesion in the fourth distal phalanx with minimal adjacent periostitis and minimal fourth digit soft tissue swelling. There was no cortical disruption or matrix calcification. Given the nonspecific findings with differential diagnoses including neoplastic or infectious conditions, further imaging was obtained. Contrast-enhanced MRI of the left hand (Fig. 3) demonstrated a low signal intensity process surrounded by a shell of cortical bone, with areas of enhancement within the lesion. There was no soft tissue component. The differential diagnosis at this time included an intraosseous glomus tumor, epidermal inclusion cyst, enchondroma, or chronic infection. Given the smooth and minimally expansile appearance, as well as no definite chondroid matrix, enchondroma was considered less likely. The absence of a significant soft tissue component and presence of intrinsic enhancement made an epidermal inclusion cyst less likely, leaving intraosseous glomus tumor or possibly chronic infection as the leading possibilities.

## Treatment and follow-up

Given the patient's persistent symptoms and location of the mass, the patient and their family agreed to undergo open surgical biopsy and curettage for both diagnostic and treatment purposes. A small open approach to the distal phalanx was performed. Curettes were used to obtain a tissue sample of the mass which was sent for both frozen and permanent pathology analysis. Intraoperative interpretation of the biopsy by our soft tissue and bone pathologist was indicative of a chondroblastoma, which was confirmed several days later with the permanent pathology studies. After confirming a benign diagnosis intraoperatively with the frozen specimen, bone graft was used to reconstruct the defect in the phalanx. Postoperatively the patient was splinted for 2 weeks, at which time sutures were removed and hand therapy was initiated. In the subsequent months, they have demonstrated continued clinical improvement. Post-operative and follow up radiographs are consistent with the history of open curettage and grafting of the site (Fig. 5). Due to the extent of distal phalangeal involvement, the plan is to continue to evaluate the patient closely for evidence of local recurrence by clinical exam and radiography. If recurrence does occur, future treatment options may include radiofrequency ablation as well as repeat surgical curettage and grafting.

## Discussion

Chondroblastoma is a rare type of benign cartilaginous bone tumor which constitutes approximately 1% of all bone neoplasms [18]. It most commonly occurs in the epiphysis of long bones in skeletally immature patients, especially the humerus, tibia, and femur. Chondroblastoma occurrence in the hand is rare, with occurrence in the phalanx being extremely rare [9]. The radiographic appearance in our case has similarities to the appearance described in long bones, including a lytic lesion with sclerotic margins and bony expansion (Figs. 2 and 3). The case we describe can be compared to the available cases presented in the literature. Garin and Wang [19] as well as Gregory et al. [7] both describe a lytic and expansile lesion involving the ring finger distal phalanx, with the exception of more periosteal new bone formation in the former. Neviaser et al. [12] described 2 patients with chondroblastoma in the middle phalanx of the hand, describing a multiloculated lytic lesion of which one appeared to have cortical perforation. Peh et al. [10] describes a metadiaphyseal lesion involving the left thumb proximal phalanx with features of an expansile lytic lesion containing thin septations and pathologic fracture. Lichtenstein and Bernstein [13] describe a lytic expansile lesion involving the second proximal phalanx. Arnander et al. [11] describe a case misdiagnosed initially as osteomyelitis following minor penetrating injury, described as middle finger distal phalanx subungual expansile lytic lesion with indistinct cortex. Kumar et al. [14] describe a mottled lucency involving the metadiaphysis of the middle phalanx ring finger.

Classic radiographic findings for chondroblastoma include a well-demarcated lytic lesion with thin sclerotic rim. These lesions tend to be small averaging 3-6 cm, and central calcifications are common with varying degrees of mineralization [16]. MRI features of the tumor matrix tend to show a lobulated rim, with low signal intensity of T1-weighted images and variable signal intensity of T2-weighted images [20]. The heterogeneous internal content is explained by the potential variability of internal contents including chondroid matrix, hypercellularity, calcifications, and hemosiderin. Chondroblastoma is commonly associated with peritumoral edema, soft tissue edema, and periostitis [21,22]. Contrast enhanced MRI can show peripheral and septal enhancement [23]. Despite classic imaging features, definitive diagnosis requires tissue sampling with histologic evaluation, especially in scenarios not fitting a classic location or epidemiologic picture. It is estimated that 15% of chondroblastomas have an aneurysmal cystic component, which has been proposed as a precursor to recurrence [24,25]. This was not observed in our case.

While the patient had a history of trauma, the persistent symptoms and imaging features warranted biopsy. Based on our patient's age and imaging findings, the differential diagnosis included intraosseous glomus tumor, epidermal inclusion cyst, and enchondroma. Other typical diagnostic considerations include giant cell tumor, aneurysmal bone cyst, and infection. An entirely intraosseous glomus tumor is exceedingly rare, with approximately 20 cases described in the literature [26]. Of these cases, the radiographic differential diagnosis included enchondroma or epithelial inclusion cyst, with correct diagnosis being revealed after surgical excision and pathologic examination. Intraosseous epidermal inclusion cysts have a male preponderance, tend to arise in the terminal phalanx of the middle finger, and have imaging features of a well-defined osteolytic lesion with sclerotic margin which may exhibit spotty calcifications [27]. Enchondroma is the most common benign tumor of the hand, and radiographically demonstrates a well-defined lytic lesion which can cause bone expansion and may have chondroid matrix [28]. On MRI enchondroma can have high signal on T2-weighted sequence, but typically show peripheral contrast enhancement as they are avascular [28].

**Fig. 4:**
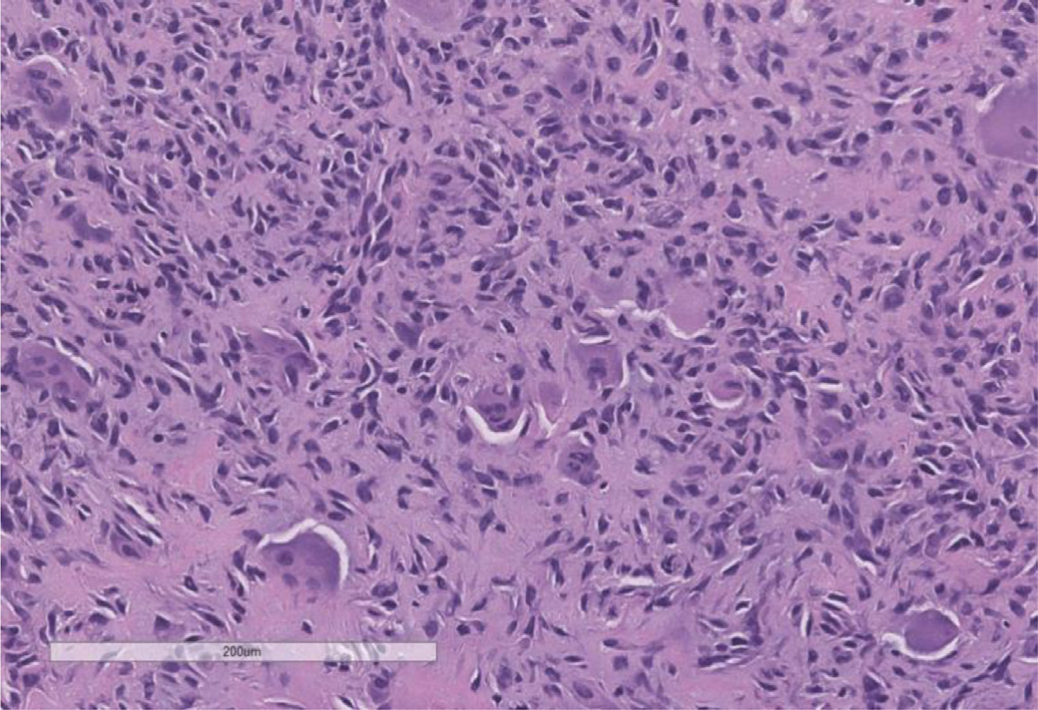
Photomicrographs of surgical biopsy tissue stained with hematoxylin and eosin demonstrate sheets of polygonal cells with ovoid or twisted nuclei, as well as few osteoclast-like giant cells.

Histologically, our case displayed expected findings for chondroblastoma and did not show any cellular atypia or mitosis (Fig. 4). The classic histological appearance for chondroblastoma is characterized by sheet-like growth of relatively undifferentiated tissue, comprised of oval polygonal chondroblast-like cells as well as scattered multi-nucleated giant cells [29]. A cartilaginous cellular matrix interspersed with areas of focal calcifications is a characteristic of the tumor. A relatively distinct pathologic finding is the presence of a region of lacy calcification, described as “chicken wire” calcification. To date, there has been no evidence in the literature of association between chondroblastoma of the phalanx and an increased risk of recurrence. While chondroblastoma can rarely metastasize to the lung, bone, and soft tissues, it does not appear to significantly affect mortality [29], [30], [31].

**Fig. 5:**
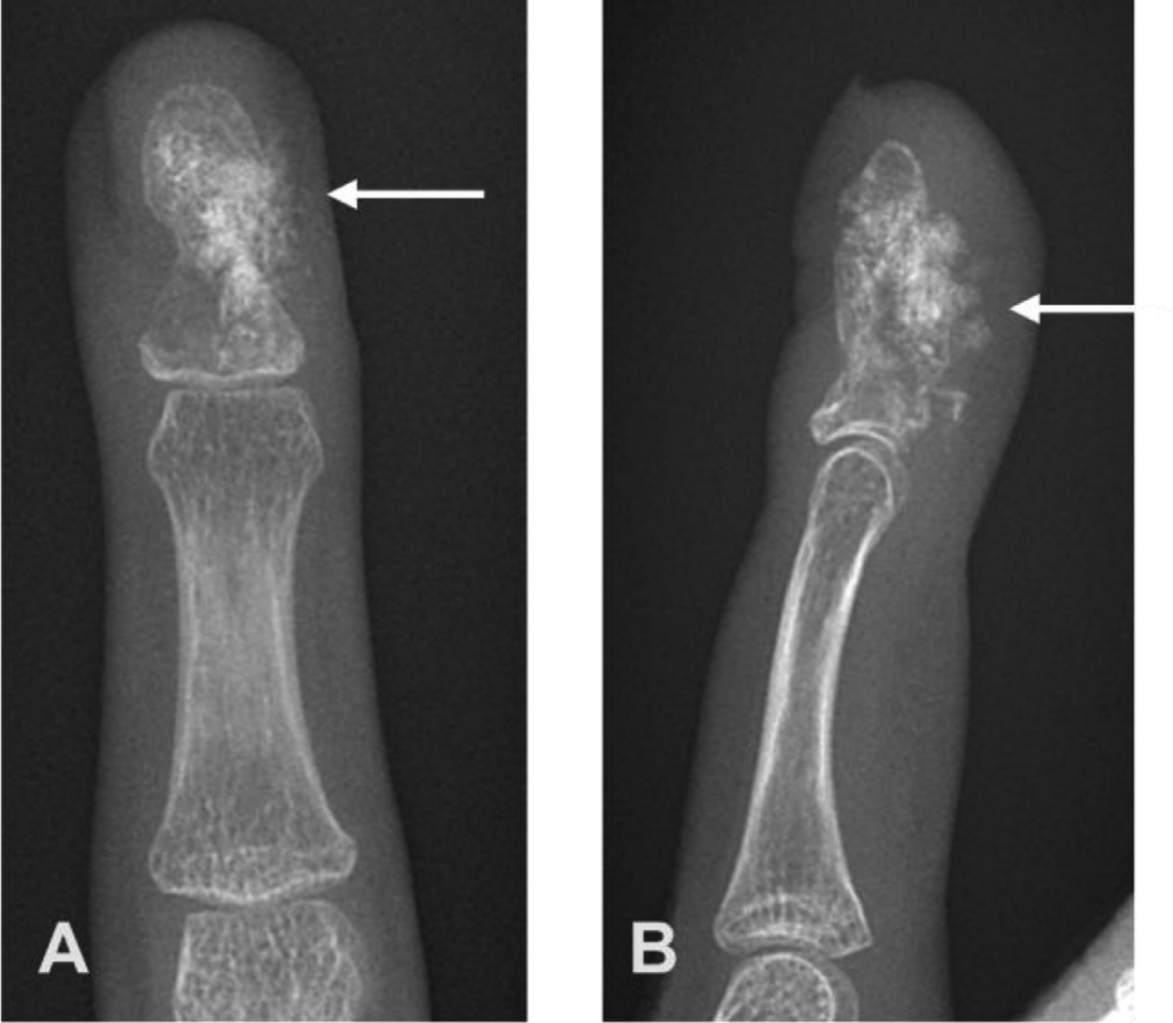
(A) PA and (B) lateral radiographs of the left hand fourth distal phalanx 10 weeks following biopsy and curettage, demonstrating postsurgical changes from open curettage and grafting (arrow).

The principal treatment choice for management of chondroblastoma is intralesional curettage and bone graft. Early and accurate diagnosis plays a crucial role in preserving hand function and preventing complications associated with the progression of the disease and potential mismanagement. Functional damage to joints can occur given the typical epiphyseal location in long bones.

## Patient consent

Written, informed consent for publication of the case report titled “A Rare Case of Chondroblastoma Involving the Phalanx” was obtained from the patient's legal guardian.
